# M3AE-Distill: An Efficient Distilled Model for Medical Vision–Language Downstream Tasks

**DOI:** 10.3390/bioengineering12070738

**Published:** 2025-07-06

**Authors:** Xudong Liang, Jiang Xie, Mengfei Zhang, Zhuo Bi

**Affiliations:** 1 School of Computer Engineering and Science, Shanghai University, Shanghai 200444, China; 2 School of Information Technology, Shanghai Jian Qiao University, Shanghai 201306, China

**Keywords:** vision–language, deep learning, knowledge distillation, pre-training

## Abstract

Multi-modal masked autoencoder (M3AE) are widely studied medical vision–language (VL) models that can be applied to various clinical tasks. However, its large parameter size poses challenges for deployment in real-world settings. Knowledge distillation (KD) has proven effective for compressing task-specific uni-modal models, yet its application to medical VL backbone models during pre-training remains underexplored. To address this, M3AE-Distill, a lightweight medical VL model, is proposed to uphold high performance while enhancing efficiency. During pre-training, two key strategies are developed: (1) both hidden state and attention map distillation are employed to guide the student model, and (2) an attention-guided masking strategy is designed to enhance fine-grained image–text alignment. Extensive experiments on five medical VL datasets across three tasks validate the effectiveness of M3AE-Distill. Two student variants, M3AE-Distill-Small and M3AE-Distill-Base, are provided to support a flexible trade-off between efficiency and accuracy. M3AE-Distill-Base consistently outperforms existing models and achieves performance comparable to the teacher model, while delivering 2.11× and 2.61× speedups during inference and fine-tuning, respectively.

## 1. Introduction

Recent advances in computational resources and the increasing availability of large-scale public datasets have significantly accelerated progress in uni-modal deep learning [1,2,3]. The perception of humans naturally integrates multi-modal information, including vision, audio signals, and language, to construct a comprehensive understanding of the environment. In the medical domain, data are generally in a multi-modal format such as chest X-ray images accompanied by corresponding clinical reports [4]. This inherent multi-modal data has driven significant interest in medical vision–language (VL) models, which aim to use multi-modal data sources to enhance modeling capabilities and comprehension [5,6]. Such models have demonstrated potential in improving diagnostic precision and reducing clinical workload through applications such as medical image–text retrieval (Med-ITR), which includes retrieving relevant textual reports given a medical image (image-to-text), and vice versa for retrieving images based on textual queries (text-to-image). Another application is medical visual question answering (Med-VQA), which aims to answer clinically relevant questions based on medical images.

In recent studies, medical VL research has trended toward increasingly complex architectures, larger parameter scales, and higher computational demands during pre-training [5,7]. For example, PubMedCLIP [6] and multi-modal masked autoencoders (M3AE) [7] contain 470 M and 347 M parameters, respectively, requiring substantial computing resources and incurring high computational costs. Although these large-scale models achieve strong performance on medical VL tasks, their deployment in real-world clinical settings remains challenging due to their computational overhead. Therefore, exploring model compression techniques for large-scale pre-trained VL models is a promising direction to improve their practicality and scalability in clinical applications.

Knowledge distillation (KD) is a widely adopted model compression technique that transfers knowledge from a large teacher model to a more compact student model [8]. The teacher–student framework is widely used in scenarios where deploying large models is impractical due to computational or memory constraints. In such cases, the teacher model can guide the student to achieve comparable performance while significantly reducing model size and inference cost. While traditional KD methods often rely on mimicking the soft outputs of the teacher, empirical studies indicate that such approaches may result in suboptimal performance, particularly in complex tasks such as medical image analysis. Consequently, recent research has focused on enhancing the distillation process to improve its effectiveness.

Although KD has been extensively studied in uni-modal medical tasks, recent works such as MSKD [9] and DSP-KD [10] have further demonstrated its effectiveness in segmentation and classification by introducing task-specific KD. However, these approaches are typically designed for single-modality settings, which limits their generalization. In the medical VL domain, KD remains relatively underexplored. For example, MHKD-MVQA [11] incorporates KD specifically for Med-VQA, making it less adaptable to other tasks. In contrast, this work introduces KD at the pre-training stage, aiming to construct a generalizable medical VL backbone that enables efficient adaptation to various downstream tasks.

Models such as M3AE [7] have shown promising results on medical VL tasks by adopting a unified pre-training framework. A concise overview of M3AE is provided in Section 2. Despite recent progress in medical VL models, two challenges limit the applicability of current methods. First, existing models adopt complex architectures with large-scale parameters, resulting in high computational costs and limited feasibility for real-world clinical deployment. Second, existing KD approaches in the medical domain are typically designed for specific tasks and uni-modal settings, making them difficult to generalize across diverse VL scenarios.

Therefore, there is a pressing need for a lightweight and generalizable medical VL model that enables efficient adaptation to various downstream tasks. To address this limitation, an efficient VL pre-trained model, M3AE-Distill, is proposed for medical VL tasks. Built upon the M3AE model, M3AE-Distill aims to effectively transfer knowledge from a high-capacity teacher model to a lightweight student model during the pre-training phase. To facilitate this KD process, three key components are developed to distill knowledge from the teacher model into a compact student model. Specifically, M3AE-Distill-Base consistently outperforms existing models and achieves performance comparable to the teacher model, while delivering 2.11× and 2.61× speedups in inference and fine-tuning, respectively. The small variant achieves even higher speedups of 3.51× and 4.83×, albeit with slightly reduced performance. The primary contributions are summarized as follows:

- KD is integrated into the pre-training pipeline by aligning both attention maps and hidden states. This distillation enables the student to approximate the teacher’s intermediate representations.
- An attention-guided masking strategy is proposed for the MIM objective. This strategy leverages attention maps from the teacher model to identify and mask semantically salient regions in the image. By reconstructing these regions, the student is encouraged to leverage complementary visual and textual features, thereby facilitating fine-grained cross-modal alignment.

## 2. Related Work

### 2.1. Vision–Language Model

Recent VL models in the general domain have shown that unified Transformer architectures can effectively integrate visual and textual information without relying on external object detectors [12,13]. These approaches establish key design principles such as joint multi-modal encoding, masked modeling, and contrastive learning, which have informed the development of domain-specific VL models.

In the medical field, VL models are adapted to better capture domain-specific semantics and structural characteristics. MedViLL [5] incorporates multi-modal attention mechanisms and shows improved alignment between medical images and text. PubMedCLIP [6], a CLIP-based model pre-trained on image–text pairs from PubMed articles, highlights the benefits of contrastive learning in medical VL settings, particularly for improving visual representations for tasks like medical VQA. Among these, M3AE [7] has emerged as a strong baseline for medical VL tasks. It is trained on approximately 300,000 image–text pairs and introduces a unified self-supervised learning framework that combines Masked Language Modeling (MLM) and Masked Image Modeling (MIM). This design enables effective representation learning from both modalities without requiring task-specific supervision. While M3AE performs well across various medical VL tasks, its large number of parameters and complex architecture present challenges for practical deployment.

### 2.2. Knowledge Distillation

KD aims to transfer knowledge from a large, complex neural network (teacher) to a smaller, more efficient network (student) [8], inspired by the way humans learn from a more knowledgeable teacher. In the vanilla KD approach, the student model is trained to mimic the soft logits produced by the teacher model. However, as these logits only capture information from the final layer, they provide weak supervision, limiting the effectiveness of the distillation process. To enhance supervision, FitNets [14] introduces an intermediate feature-based distillation approach, where the student model learns from the teacher’s feature attention maps, providing richer guidance beyond output logits. For Transformer-based architectures, self-attention plays a crucial role in capturing long-range dependencies between tokens. Leveraging this insight, TinyBERT [15] proposes an attention-based distillation mechanism to transfer the teacher’s linguistic and structural knowledge to the student model. In this study, the teacher–student framework is adopted to retain the strong cross-modal alignment and semantic reasoning capabilities learned by the teacher, while enabling efficient deployment through a lightweight student.

## 3. Method

The proposed M3AE-Distill model is a distilled version of M3AE, incorporating CLIP-ViT (ViT-B/16) and RoBERTa as uni-modal encoders. The image encoder consists of 12 Transformer layers with a 16×16 patch size and is initialized from CLIP pre-trained weights. Multi-modal feature fusion is achieved through cross-modal encoders.

**Figure 1:**
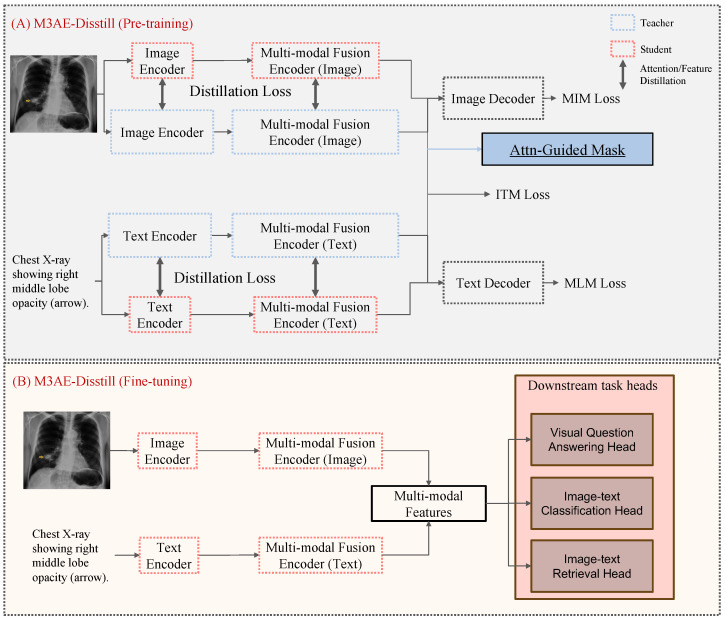
Overview of the M3AE-Distill model.

The training process consists of two stages, as illustrated in Figure 1. In Stage 1 (Figure 1A), the student model is trained on pre-training tasks under the guidance of the teacher model. This stage includes two key components: (1) both attention and hidden state distillation are employed to transfer knowledge from the teacher model to the student, facilitating effective representation learning; and (2) an attention-guided masking strategy is applied to identify and mask semantically salient regions in the image, promoting the fine-grained alignment of visual and textual features. In Stage 2 (Figure 1B), the student model is fine-tuned for downstream medical VL tasks, including Med-VQA, Med-CLS, and Med-ITR. The joint representations learned during pre-training are utilized, while the teacher model is not involved in this stage. This setup highlights the transferability and efficiency of the student model.

### 3.1. Pre-Training Tasks

The M3AE-Distill model was pre-trained with three tasks: MLM, MIM, and ITM. The objectives of these tasks are outlined below.

#### 3.1.1. Masked Language Modeling

MLM is a self-supervised objective that enables the model to learn word features by predicting masked tokens within a given text. In VL models, incorporating visual features into MLM facilitates enhanced textual understanding and cross-modal feature integration.

Given an image–text pair (x,t), where *x* represents the image and *t* denotes the text, the text *t* is tokenized using a RoBERTa tokenizer with a maximum sequence length of 64 tokens, resulting in $t=\{w_{1},w_{2},\ldots ,w_{n}\}$. A total of 15% of the tokens were randomly and uniformly selected for potential masking. This randomness helps the model learn robust contextual representations and avoid overfitting. To avoid over-reliance on local context (i.e., the tendency to focus only on nearby tokens and ignore long-range dependencies), 80% of the masked tokens were replaced with the special token [MASK], 10% were substituted with random words, and the remaining 10% remained unchanged. The processed text $\tilde{t}=\left\{w_{1},w_{2},\left[MASK\right],\left[MASK\right],w5,\ldots ,w_{n}\right\}$ was encoded by RoBERTa, while the image was processed by CLIP-ViT. These features were fused via a cross-modal encoder to produce a joint representation $z_{x\tilde{t}}$. A fully connected layer followed by a softmax activation was used to produce a probability distribution over the entire vocabulary for each masked token, and the model was optimized using the cross-entropy loss:

(1) $$L_{\mathrm{mlm}}=E_{(x,\tilde{t})∼D}H\left(y^{msk-t},p^{msk-t}\left(x\tilde{t}\right)\right),$$

where $y^{msk-t}$ denotes the ground-truth tokens for the masked positions, and $p^{msk-t}\left(x\tilde{t}\right)$ represents the predicted probability distribution over the vocabulary.

#### 3.1.2. Masked Image Modeling

Inspired by the success of the MLM task and aiming to enhance the model’s ability to learn visual context and spatial relationships, MIM was designed to predict masked regions within an image. Here, spatial relationships refer to structural dependencies among image patches, modeled via self-attention.

The image *x* was partitioned into non-overlapping 16 × 16 pixel patches, denoted as $x=\{x_{1},x_{2},\ldots ,x_{m}\}$, where *m* is the number of patches. Masking was performed at the patch level, where 75% of the patches were randomly selected and masked. The remaining unmasked sequence $\tilde{x}=\left\{x_{1},x_{3},x_{7},\ldots \right\}$ was fed to CLIP-ViT to generate visual features $z_{\tilde{x}}$. Random masking encourages the model to capture contextual dependencies and prevents overfitting to fixed spatial patterns. Thus, the final cross-modal representation was obtained by concatenating the visual features and textual features, which were then processed by a cross-modal encoder to generate joint features. A decoder was subsequently used to predict the missing patches based on these joint features. The objective was to minimize the reconstruction error, using mean squared error (MSE) between the predicted and original image patches:

(2) $$L_{\mathrm{mim}}=E_{(\tilde{x},t)∼D}H\left(y^{msk-x},p^{msk-x}\left(\tilde{x}t\right)\right),$$

where $y^{msk-x}$ represents the ground-truth pixels of the masked patches, and $p^{msk-x}\left(\tilde{x}t\right)$ denotes the predicted pixel values.

#### 3.1.3. Image–Text Matching

To enhance cross-modal understanding ability, ITM loss was employed to predict the semantic alignment between image and text. Semantic alignment ensures that the paired image and text representations correspond at a conceptual level. This is critical for downstream tasks such as Med-ITR and Med-VQA.

The training samples for ITM consist of both positive and negative pairs. Positive samples are matched image–text pairs from the dataset, while negative samples are generated by substituting either the image or the text with a randomly selected alternative. This random selection is essential as it introduces diversity and unpredictability into the negative pairs, preventing the model from overfitting to specific patterns and encouraging it to learn robust semantic relationships. Then, the model computes the above complete image–text pairs to obtain cross-modal representation, followed by a classification head to predict the semantic alignment probability. The ITM loss is formulated using binary cross-entropy:

(3) $$L_{\mathrm{itm}}=E_{(x,t)∼D}H\left(y^{itm},p^{itm}\left(xt\right)\right),$$

where $y^{itm}$ indicates whether the image–text pair is correctly matched (1 for positive pairs, 0 for negative pairs).

Finally, the overall pre-training loss of M3AE-Distill is defined as

(4) $$L_{\mathrm{pre}-\mathrm{training}}=L_{\mathrm{mlm}}+L_{\mathrm{mim}}+L_{\mathrm{itm}}$$

### 3.2. Knowledge Distillation

KD is a widely adopted model compression technique designed to transfer knowledge from a high-capacity teacher model to a more compact student model, thereby improving computational efficiency. In this study, KD is applied during the pre-training stage, rather than during downstream task-specific fine-tuning, allowing the student to learn transferable VL representations. The student model adopts a pure Transformer architecture to model uni-modal and multi-modal features. In this context, hidden states encode rich semantic representations from visual and textual modalities, while attention maps capture structural and alignment patterns between modalities. Following the approach in [16], both hidden layer distillation and attention map distillation are employed to facilitate effective knowledge transfer from the teacher to the student model.

#### 3.2.1. Hidden Layer Distillation

Hidden layer distillation [8] minimizes the discrepancy between intermediate feature representations of the teacher and student models, enabling the student to acquire deep representations from the teacher. Formally, let $H_{T}\in R^{L\times d}$ and $H_{S}\in R^{l\times d}$ denote the intermediate feature representations of the teacher and student models, respectively, where *L* and *l* represent the number of layers in each model, and *d* denotes the hidden layer dimension. *l* hidden layers from the teacher model are uniformly sampled to align with all student layers. The similarity between corresponding layers is enforced using the MSE loss:

(5) $$L_{\mathrm{hid}}=\sum_{i=1}^{l}\mathrm{MSE}\left(H_{i}^{S},H_{m(i)}^{T}\right),$$

where m(i) indicates the index of the teacher layer selected to align with the *i*-th student layer via uniform sampling across the teacher’s depth.

#### 3.2.2. Attention Distillation

Attention distillation [15] encourages the student model to mimic the self-attention distributions of the teacher model, thereby improving its representational capacity. In Transformer-based architectures, the attention weight matrix A is computed as

(6) $$A=\mathrm{softmax}\left(\frac{QK^{⊤}}{\sqrt{d_{k}}}\right),$$

where $d_{k}$ denotes the key dimension, and Q and K represent the query and key matrices, respectively. To ensure that the student model effectively learns the teacher’s attention patterns, attention maps from the student model are aligned with those from the teacher model. Specifically, teacher layers are evenly sampled by selecting one layer every few layers, and each student layer is aligned with the last layer in the corresponding interval. The attention distillation loss is defined as

(7) $$L_{\mathrm{attn}}=\frac{1}{h}\sum_{j=1}^{l}\sum_{i=1}^{h}\mathrm{MSE}\left(A_{S}^{\left(i,j\right)},A_{T}^{\left(i,m^{\prime }\left(j\right)\right)}\right),$$

where *h* denotes the number of attention heads, $A_{S}^{\left(i,j\right)}$ represents the attention matrix of the *i*-th head at the *j*-th student layer, and $A_{T}^{\left(i,m^{\prime }\left(j\right)\right)}$ denotes the attention matrix from the selected teacher layer corresponding to the *j*-th student layer.

For the Small model, a 3:1 mapping is applied in the uni-modal encoders, and a 6:1 mapping is used in the multi-modal encoder. For the Base model, a 2:1 mapping is used for the uni-modal encoders, and a 3:1 mapping is applied in the multi-modal encoder.

Thus, the distillation loss is summarized as

(8) $$L_{\mathrm{distill}}=L_{\mathrm{hid}}+L_{\mathrm{attn}}$$

### 3.3. Attention-Guided Masked Image Modeling

Medical images are characterized by low contrast and high structural similarity between different tissues, which together make the extraction of regions of interest (ROIs) particularly challenging [17]. These characteristics reduce the effectiveness of conventional MIM tasks in the medical domain. Ideally, the MIM task should prioritize these ROIs to encourage the model to focus on both local image features and textual information during reconstruction. But in the medical domain, the conventional MIM strategy is suboptimal, as the surrounding areas are large and are masked with a high probability. Consequently, the MIM task may degenerate into relying on visual features to predict masked pixel values, thereby diminishing the contribution of textual information.

Recent studies [18,19] have demonstrated that attention scores in Transformer-based models effectively identify salient image regions and enhance model interpretability. Motivated by this observation, an attention-guided MIM strategy is introduced. While AttMask [20] demonstrates the effectiveness of attention-guided masking in uni-modal image modeling, its direct application to VL models remains limited. Inspired by the core idea of AttMask, the proposed method extends this concept to the VL setting by leveraging attention scores from both teacher and student models to guide selective masking and reconstruction. Furthermore, by progressively increasing the masking ratio on key regions during training, the approach encourages fine-grained cross-modal alignment between visual and textual representations.

**Figure 2:**
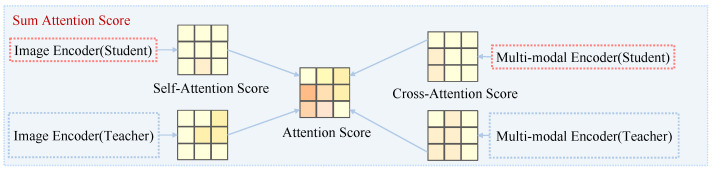
Computation of the aggregated attention score matrix.

**Figure 3:**
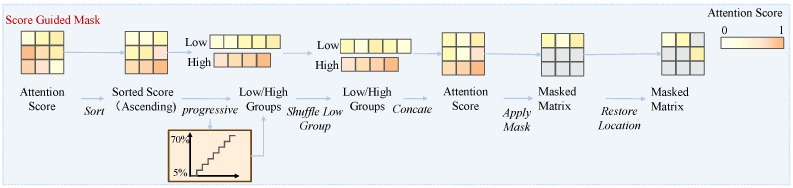
Attention-guided masking strategy.

The proposed strategy consists of two stages, as illustrated in Figure 2 and Figure 3. The first stage computes an aggregated attention matrix by combining attention maps from both the teacher and student models across uni-modal and multi-modal branches. The second stage uses this matrix to progressively generate masks that prioritize high-attention areas during training, enabling the model to gradually enhance cross-modal alignment.

#### 3.3.1. Stage 1: Attention Score Matrix Computation

As shown in Figure 2, given each image–text pair (x,t), both the teacher and the student model are used to extract uni-modal and multi-modal attention maps. Let *N* denote the number of image patches. Four attention matrices are computed: uni-modal attention from the student model $A_{S}^{uni}\in R^{N\times N}$, uni-modal attention from the teacher model $A_{T}^{uni}\in R^{N\times N}$, cross-modal attention from text to image in the student model $A_{S}^{mul}\in R^{N\times N}$, and the corresponding cross-modal attention in the teacher model $A_{T}^{mul}\in R^{N\times N}$. These four matrices are summed and then normalized to produce the final attention guidance matrix:

(9) $$A=\mathrm{Normalize}\left(A_{S}^{uni}+A_{T}^{uni}+A_{S}^{mul}+A_{T}^{mul}\right)\in {\left[0,1\right]}^{N\times N}$$

This matrix reflects the relative importance of each image region to the model’s overall judgment, with higher attention values indicating more critical regions.

#### 3.3.2. Stage 2: Attention-Guided Progressive Mask Generation

As shown in Figure 3, based on the attention matrix *A* obtained in Stage 1, this stage performs progressive masking over the image to guide the fine-grained alignment between the image and text. The procedure is as follows:

1. Sort the attention scores in ascending order to obtain the sorted attention matrix $A_{\mathrm{sort}}$.
2. Progressively adjust the proportion *r* of high-attention group based on training progress:
  (10) $$r=r_{start}-\left(r_{start}-r_{end}\right)\cdot \frac{step}{max\_steps},$$
  where $r_{start}=0.95$ means that at the beginning of training, only the top 5% of patches are treated as high-attention. Over the training phase, *r* decreases to $r_{end}=0.3$, meaning up to 70% of patches may be included in the high-attention group. In this way, the threshold becomes more inclusive as training progresses, allowing the model to gradually expand its focus beyond the most salient regions.
3. Divide the patches into high-attention and low-attention groups according to *r*. All patches in the high-attention group are treated as key areas and are masked. For the low-attention group, a proportion of 75%−r is randomly masked to introduce noise and enhance robustness.
4. The two masked groups are then recombined and restored to their original order, yielding the final binary mask matrix $M\in {\left\{0,1\right\}}^{N\times N}$.

This strategy enables the mask to initially concentrate on a small set of key regions, encouraging the model to learn localized discriminative features. As training progresses, the mask range expands to cover more high score areas, promoting fine-grained alignment.

#### 3.3.3. Case Study

**Figure 4:**
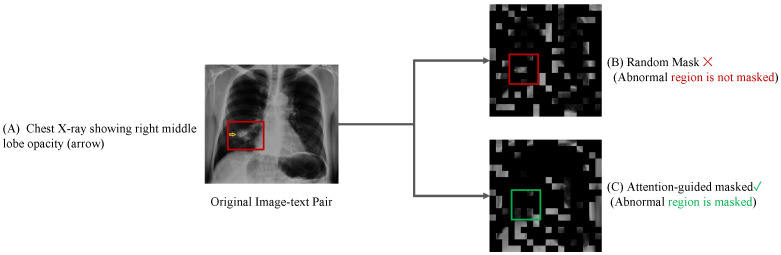
Comparison of random masking and attention-guided masking strategies.

As depicted in Figure 4, for the image–text pair (A), the red bounding box highlights the abnormal region. The abnormal region is only partially masked under the conventional masking strategy (B). In contrast, the proposed attention-guided masking strategy (C) adaptively selects mask regions based on cross-modal attention scores, ensuring that the abnormal region is more likely to be masked while reducing the masking ratio for background areas.

The overall pre-training loss is formulated as

(11) $$L_{\mathrm{total}}=L_{\mathrm{pre}-\mathrm{training}}+L_{\mathrm{distill}}$$

## 4. Experiments

### 4.1. Pre-Training Datasets

Following M3AE [7], the ROCO [21] and MedICaT [22] datasets were utilized for pre-training. Both datasets are derived from PubMed and are widely adopted in medical VL research. The ROCO dataset consists of approximately 81,000 radiology image–text pairs, covering modalities such as CT, MRI, X-ray, PET, ultrasound, and angiography. Each image is paired with a caption, and many also include metadata such as UMLS Concept Unique Identifiers (CUIs) and semantic types, enabling structured semantic alignment. The MedICaT dataset contains around 217,000 medical figures extracted from over 130,000 biomedical papers. About 93% of the images are medical (e.g., radiology, histology, endoscopy), and 75% are compound figures. Each figure includes a caption with an average length of 74 tokens, and 74% have additional inline textual references (average 67 tokens), providing rich multi-modal context.

### 4.2. Downstream Datasets

To evaluate the model’s performance, experiments were conducted across three medical VL tasks using five datasets.

For the Med-VQA task, the VQA-RAD [23], SLAKE [24], and VQA-2019 [25] datasets were employed, with answer accuracy serving as the evaluation metric:

(12) $$\mathrm{Score}=\frac{\mathrm{Correct}\;\mathrm{Predictions}}{\mathrm{Total}\;\mathrm{Predictions}}.$$

For the Med-CLS task, performance was assessed on the MELINDA [26] dataset using the accuracy metric:

(13) $$Acc=\frac{TP+TN}{TP+TN+FP+FN},$$

where TP represents the number of true positives, TN represents the number of true negatives, FP represents the number of false positives, and FN represents the number of false negatives.

For the Med-ITR task, evaluations were conducted on the ROCO [21] dataset. This task includes both image-to-text retrieval (I2T), which retrieves the most relevant textual description given an input image, and text-to-image retrieval (T2I), which retrieves the most relevant image based on a textual query. Performance is measured using Recall@K (R@K), which indicates the proportion of queries for which the correct match appears within the top *K* retrieved results:

(14) $$\mathrm{Recall}@K=\frac{\mathrm{Queries}\;\mathrm{with}\;\mathrm{Correct}\;\mathrm{Match}\;\mathrm{in}\;\mathrm{Top}\;K}{\mathrm{Total}\;\mathrm{Queries}}.$$

### 4.3. Implementation Details

#### 4.3.1. Experiment Settings

All experiments were conducted using PyTorch-1.9.0. During pre-training, images were resized to 288×288, and text sequences were either truncated or padded to 64 tokens. For fine-tuning, images were resized to 384×384, except for the image–text retrieval task, where a resolution of 288×288 was maintained. All reported metrics were evaluated on the test sets.

AdamW was used as the optimizer for all experiments. The learning rate was set to 1 × 10^−5^, and a linear scheduler with a 10% warm-up ratio was applied, gradually decreasing the learning rate to zero after warm-up. Both pre-training and fine-tuning were conducted on two NVIDIA RTX 3090 GPUs with a total batch size of 32.

#### 4.3.2. Teacher–Student Model Comparison

As shown in Table 1, the two student models designed in this study differ from the teacher model in both architectural complexity and parameter scale, enabling flexible deployment under varying computational resource constraints.

The teacher model adopts a 12-layer Transformer for both image and text encoders, each accompanied by a 6-layer multi-modal interaction module. Specifically, two student variants, M3AE-Distill-Small and M3AE-Distill-Base, are developed with different parameter budgets. The Small version represents the most lightweight configuration, retaining only 5 image encoder layers, 4 text encoder layers, and a single-layer multi-modal module, resulting in a total of 141.6 M parameters. This design prioritizes fast inference and low-resource applicability, while preserving essential representational capacity. The Base version moderately increases the network depth to improve modeling capacity, resulting in 188.8 M parameters—approximately 55% of the teacher model’s size. This version aims to strike a balance between efficiency and performance, particularly in scenarios where moderate computational resources are available.

### 4.4. Results and Discussion

#### 4.4.1. Medical VQA Results

The experimental results are presented in Table 2. MEVF is pre-trained on approximately 10,000 samples, whereas CPRD is trained on a larger dataset of roughly 20,000 samples. CPRD achieves overall scores of 67.80 and 81.10 on the VQA-RAD and SLAKE datasets, respectively, outperforming MEVF by 1.7 and 2.5 points. These improvements are attributed to the increased scale of pre-training data and the more complex pre-training tasks. Notably, PubMedCLIP, pre-trained on the ROCO dataset comprising about 81,000 samples, achieves an overall score of 72.10 on the VQA-RAD, while exceeding CPRD by 4.3 points, but showing a slight decrease of 1.0 points on the SLAKE dataset.

M3AE demonstrates the best performance among all baselines, achieving 77.01, 83.25, and 79.87 on VQA-RAD, SLAKE, and VQA-2019, respectively. M3AE-98% maintains 98% of the full model’s performance across all evaluation metrics.

The proposed M3AE-Distill models achieve strong results while maintaining compact model sizes. Specifically, M3AE-Distill-Base delivers competitive performance, ranking second on both VQA-RAD (75.55) and SLAKE (82.16), and achieving 78.46 on VQA-2019. Meanwhile, M3AE-Distill-Small yields reasonable performance (73.45 on VQA-RAD and 80.32 on SLAKE) with substantially fewer parameters.

Compared to its teacher model M3AE, M3AE-Distill-Base reduces the parameter count by approximately 150 M while incurring only marginal performance degradation. The accuracy gap remains minimal on SLAKE and VQA-2019 (82.16 vs. 83.25 and 78.46 vs. 79.87, respectively), and it generally performs better than M3AE-98%. Although M3AE-Distill-Small underperforms the Base variant, it still surpasses earlier models such as CPRD-BAN and PubMedCLIP.

#### 4.4.2. Medical Classification Results

Table 3 reports the performance of models fine-tuned on the MELINDA dataset. As shown, uni-modal models perform relatively poorly, with ResNet-101 and RoBERTa achieving 63.84 and 74.60 accuracy, respectively. In contrast, multi-modal models generally achieve superior results.

Among all evaluated models, M3AE achieves the highest accuracy (78.50), followed closely by M3AE-Distill-Base with 77.37, and M3AE-98% with 76.93. M3AE-Distill-Small achieves 74.31 accuracy, slightly lower than other multi-modal models. This demonstrates its effectiveness in resource-constrained scenarios, offering a favorable balance between model compactness and performance.

Overall, both variants of M3AE-Distill exhibit strong performance on the MELINDA classification task. M3AE-Distill-Base provides a competitive alternative to the full M3AE model with a significant reduction in parameter count, while M3AE-Distill-Small offers a lightweight option suitable for low-resource deployment.

#### 4.4.3. Medical Retrieval Results

The retrieval results on the ROCO dataset are summarized in Table 4. Among all evaluated methods, M3AE achieves the best performance across all retrieval metrics. These results significantly outperform prior models such as ViLT and PubMedCLIP, demonstrating the effectiveness of multi-modal pre-training with large-scale datasets.

The proposed M3AE-Distill-Base ranks second overall, achieving R@1 scores of 14.36 (T2I) and 14.80 (I2T), along with competitive results at R@5 and R@10. Despite its reduced parameter count, it retains most of the retrieval performance of the full M3AE model. In contrast, M3AE-Distill-Small shows a marked drop in retrieval task (e.g., R@1 of 4.05 for T2I), despite its acceptable performance on Med-VQA and Med-CLS tasks. This contrast suggests that retrieval tasks are more sensitive to representation capacity and cross-modal alignment quality. While the Small variant strikes a good balance for simpler tasks like VQA and classification, it appears insufficient for retrieval, which demands stronger fine-grained modeling across modalities.

#### 4.4.4. Efficiency Comparison Results

Table 5 compares the training and inference efficiency of different models. As expected, the student models demonstrate substantial improvements in both throughput and CPU inference latency compared to the teacher model.

M3AE-Distill-Small achieves the highest efficiency, with a training throughput of 409.81 pairs/s and inference throughput of 417.29 pairs/s, representing a 4.83× and 3.51× speedup over the teacher model, respectively. Additionally, its CPU inference latency is reduced to 100 ms per sample—about 3.7× faster than M3AE, which makes it highly suitable for deployment in resource-constrained environments. M3AE-Distill-Base also delivers notable gains, achieving 2.61× training and 2.11× inference speedup, with a CPU latency of 208 ms (1.78× faster).

These results demonstrate that both student variants offer considerable efficiency advantages, with Small prioritizing lightweight deployment and Base offering a more balanced trade-off between performance and speed.

#### 4.4.5. Ablation Studies

#### Module Ablation

To assess the effectiveness of the proposed modules, models are first pre-trained on pre-training datasets and then fine-tuned on the SLAKE dataset. The results are shown in Table 6, where M3AE-Distill-Base is selected as the comparison.

ID 0 corresponds to the M3AE model, serving as an upper-bound reference. ID 1 represents the student model trained with pre-training only, yielding an overall accuracy of 81.26. In ID 2, KD is introduced, resulting in a performance gain to 81.64, demonstrating the effectiveness of distillation in improving student model alignment with the teacher. ID 3 incorporates the proposed attention-guided masking strategy, which further enhances the overall performance to 82.16, while achieving the highest open metric (80.57), highlighting the effectiveness of this module.

#### Attention Score Source

To evaluate the influence of attention score composition on the masking strategy, three configurations are compared, as shown in Table 7. In the Student-only setting, attention maps are derived solely from the student model; in the Teacher-only setting, they are derived from the frozen teacher model; and in the Student+Teacher setting, they are derived from the element-wise summation of both.

The highest overall accuracy (82.16) is obtained when combining student and teacher attention, indicating that attention information from both models contributes complementary guidance for identifying salient visual regions. In contrast, using attention scores from either the student or teacher alone resulted in slightly lower performance (81.36 and 81.40, respectively), suggesting that a single-source attention signal may be insufficient for optimal mask generation.

#### 4.4.6. Case Study

#### Feature Distribution Visualization via PCA

**Figure 5:**
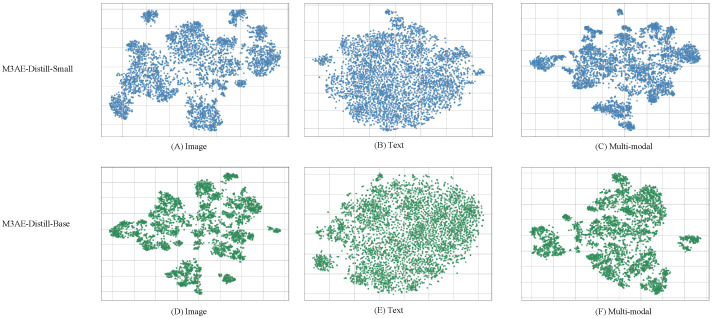
PCA visualizations of image, text, and multi-modal embeddings.

To qualitatively assess the representation quality of the student models, 5000 samples from the ROCO dataset are selected, and their feature embeddings from the image encoder, text encoder, and multi-modal encoder are visualized using PCA, as shown in Figure 5.

Clearer cluster boundaries are observed in the image encoder of the Base model, whereas the Small model shows more redundant and dispersed clusters, suggesting weaker visual representation modeling. In the text encoder, both models produce similarly compact distributions, indicating that each is capable of effectively capturing semantic information from the text modality. For the multi-modal encoder, the Base model exhibits well-separated clusters, reflecting better cross-modal alignment and semantic integration, while the Small model displays a more diffuse pattern, revealing limitations in joint representation learning.

**Figure 6:**
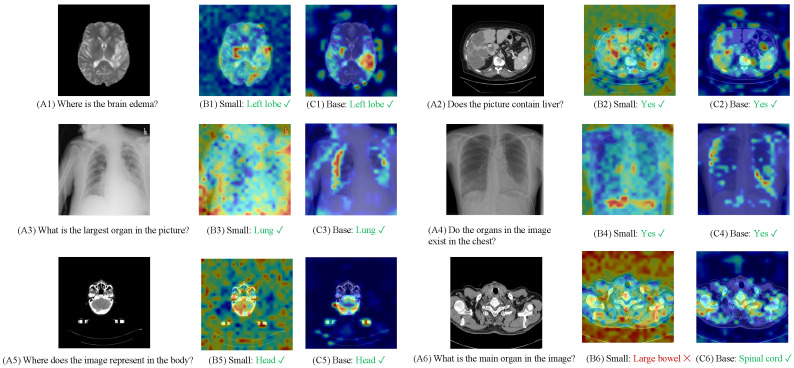
Case study on the SLAKE dataset. Attention maps highlight the regions in the image attended to by the model in response to the question.

Six samples from the SLAKE dataset are selected to assess the effectiveness of M3AE-Distill. The cross-attention weights from text to image are employed to visualize the attention distribution in the image branch, as illustrated in Figure 6, where (A) shows the input image and question, (B) shows the prediction from the Small model, and (C) shows the prediction from the Base model.

Across all cases, both models demonstrate reasonable comprehension and grounding capabilities, while the Base model generally exhibits more focused and anatomically accurate attention distributions. For example, in the last sample, the Small model incorrectly identifies the “large bowel” due to diffuse attention, whereas the Base model correctly locates and answers the “spinal cord” with a more precise activation map.

## 5. Conclusions

This study builds on the M3AE medical VL model and introduces M3AE-Distill, an efficient framework for medical VL tasks, including VQA, classification, and image–text retrieval. Although M3AE benefits from large-scale pre-training datasets and extensive tunable parameters, its deployment is constrained by the substantial computational demands. To address this limitation, KD is employed to compress M3AE into a lightweight model, M3AE-Distill. To enhance knowledge transfer from teacher to student, an attention-guided masking strategy is developed.

The experimental results demonstrate that M3AE-Distill outperforms prior medical VL models. It attains performance comparable to that of the teacher model while significantly enhancing computational efficiency.

For future work, more advanced KD techniques will be explored to further compress VL models without sacrificing performance. Furthermore, improving the robustness and generalization of medical VL models to better adapt to diverse real-world medical datasets remains a key direction for future research.

### Limitations and Prospects

Despite its demonstrated effectiveness across several medical VL tasks, the proposed M3AE-Distill framework still faces certain limitations. One key limitation lies in the nature of the pre-training data, which consist exclusively of 2D medical images, such as X-rays and 2D slices of CT or ultrasound. As a result, the model may not effectively capture the spatial continuity and contextual depth required for interpreting full 3D volumetric data, such as complete CT or MRI scans. Addressing this limitation may require adapting the model architecture to incorporate 3D spatial representations or designing pre-training strategies that account for volumetric information. In addition, the current framework assumes access to clean and fully aligned image–text pairs. Extending the model to handle weak supervision, noisy annotations, or partially missing modalities would further increase its practical applicability in clinical settings.

## Figures and Tables

**Table 1:** Comparison of model architectures and parameter counts.

Model Name	Uni-Modal (Layers L/Params M)		Multi-Modal (Layers L/Params M)		Total Params (M)
	Image	Text	Image	Text	
Teacher (M3AE)	12/104 M	12/124 M	6/56.7 M	6/56.7 M	341.4 M
Student (Small)	5/54.7 M	4/67.9 M	1/9.5 M	1/9.5 M	141.6 M
Student (Base)	7/68.9 M	6/82.1 M	2/18.9 M	2/18.9 M	188.8 M

**Table 2:** VQA results on VQA-RAD, SLAKE and VQA-2019 datasets (**Best**; Second-Best).

Methods	VQA-RAD			SLAKE			VQA-2019
	Open	Closed	Overall	Open	Closed	Overall	Overall
MEVF-SAN [27]	49.20	73.90	64.10	75.30	78.40	76.50	68.90
MEVF-BAN [27]	49.20	77.20	66.10	77.80	79.80	78.60	77.86
CPRD-BAN [28]	52.50	77.90	67.80	79.50	83.40	81.10	-
PubMedCLIP [6]	60.10	80.00	72.10	78.40	82.50	80.10	-
M3AE [7]	**67.23 **	**83.46**	**77.01**	**80.31**	**87.82**	**83.25**	**79.87**
M3AE-98% [7]	65.89	81.79	75.47	78.70	86.06	81.59	79.27
M3AE-Distill-Small	64.25	79.49	73.45	78.17	83.65	80.32	74.07
M3AE-Distill-Base	65.92	81.87	75.55	**80.57**	84.62	82.16	78.46

**Table 3:** Classification results on MELINDA dataset. (**Best**; Second-Best).

Methods	Modality	Acc
ResNet-101 [1]	Image	63.84
RoBERTa [2]	Text	74.60
NLF [26]	Image + Text	76.60
SAN [29]	Image + Text	72.30
M3AE [7]	Image + Text	**78.50**
M3AE-98% [7]	Image + Text	76.93
M3AE-Distill-Small	Image + Text	74.31
M3AE-Distill-Base	Image + Text	77.37

**Table 4:** Image–text retrievals results on ROCO dataset. (**Best**; Second-Best).

Methods	T2I			I2T		
	R@1	R@5	R@10	R@1	R@5	R@10
ViLT [12]	9.75	28.95	41.40	11.90	31.90	43.20
PubMedCLIP * [6]	8.61	26.73	38.49	8.16	25.78	38.24
M3AE * [7]	**16.96**	**46.47**	**61.33**	**17.65**	**46.40**	**60.95**
M3AE-Distill-Small	4.05	14.96	24.36	5.40	19.95	29.20
M3AE-Distill-Base	14.36	37.17	51.23	14.80	36.90	50.20

* denotes our reproduced results.

**Table 5:** Training and inference efficiency comparison of different models.

Model Variant	Training (pairs/s)	Inference (pairs/s)	CPU Inference (ms/pair)	Speedup (Train/Inference/CPU)
Teacher (M3AE)	84.77	119.07	370	1.00/1.00/1.00
Student (Small)	409.81	417.29	100	4.83/3.51/3.70
Student (Base)	221.61	251.47	208	2.61/2.11/1.78

**Table 6:** Ablation experiments of employed modules (**Best**; Second-Best).

ID	Strategy	Open	Close	Overall
0	M3AE (Teacher)	80.31	**87.82**	**83.25**
1	Pre-training	79.10	84.62	81.26
2	Pre-training + KD	79.26	85.37	81.64
3	Pre-training + KD + Attention Mask	**80.57**	84.62	82.16

**Table 7:** Ablation experiments of attention score source (**Best**; Second-Best).

Attention Score Type	Open	Close	Overall
Student-only	80.03	83.41	81.36
Teacher-only	80.03	83.53	81.40
Student + Teacher	**80.57**	**84.62**	**82.16**
